# Conflict resolution styles and skills and variation among medical students

**DOI:** 10.1186/s12909-023-04228-x

**Published:** 2023-04-14

**Authors:** Rathnayaka M. Kalpanee D. Gunasingha, Hui-Jie Lee, Congwen Zhao, Alison Clay

**Affiliations:** 1 Department of Surgery, Uniformed Services University of Health Sciences and Walter Reed National Military Medical Center, Bethesda, MD USA; 2 Department of Biostatistics and Bioinformatics, School of Medicine, Duke University, Durham, NC USA; 3 Department of Medicine, School of Medicine, Duke University, Durham, NC USA; 4 Department Medical Education, School of Medicine, Duke University, Durham, NC USA

**Keywords:** Conflict resolution, Undergraduate medical education, Entrustability, Conflict resolution style

## Abstract

**Background:**

Conflict is inevitable on healthcare teams, yet few professional school curricula teach or assess conflict resolution skills. Little is known about the variation in conflict resolution styles across medical students and how these styles might impact conflict resolution skills.

**Methods:**

This is a prospective, single blinded, group randomized quasi experimental trial to assess the impact of knowing one’s own conflict resolution style on conflict resolution skills in a simulated encounter. Graduating medical students completed a mandatory conflict resolution session with standardized patients acting as nurses during a transition to residency course. Coaches reviewed videotapes of the simulation, focusing on students’ skills with negotiation and emotional intelligence. Retrospectively, we assessed the impact of the students knowing their conflict resolution style prior to simulation, student gender, race, and intended field of practice on conflict resolution skills as judged by coaches.

**Results:**

One hundred and eight students completed the simulated conflict session. Sixty-seven students completed the TKI before the simulated patient (SP) encounter and 41 after. The most common conflict resolution style was accommodating (n = 40). Knowing one’s conflict resolution style in advance of the simulation and one’s identified race/ethnicity did not impact skill as assessed by faculty coaches. Students pursuing diagnosis-based specialties had higher negotiation (*p* = 0.04) and emotional quotient (*p* = 0.006) scores than those pursuing procedural specialties. Females had higher emotional quotient scores (p = 0.02).

**Conclusions:**

Conflict resolution styles vary among medical students. Male gender and future practice in a procedural specialty impacted conflict resolution skills but knowing conflict resolution style did not.

**Supplementary Information:**

The online version contains supplementary material available at 10.1186/s12909-023-04228-x.

## Introduction

The healthcare environment is a complex system of relationships. The interplay amongst different professionals, staff, and trainees allows for the successful care of patients most of the time, but with daily stressors, it can also be a source of conflict. While conflict may improve the quality of decisions and enhance the understanding of diverse viewpoints,[1–3] relationship conflicts negatively impact productivity, creativity, satisfaction, and task-related effort [1, 4]. Rudeness in the NICU decreased diagnostic and procedural performance [5]. Conflict also contributes to burnout [6]. In healthcare, poor working relationships correlated to higher levels of burnout in critical care nurses, physicians, and general hospital staff [7–9]. Personal conflict style and emotional intelligence have been found to have an impact on work environment, and subsequently, burnout [6, 10, 11].

As a result, many professional training programs, including law and business schools, provide training in conflict resolution [12–14]. The need for formal training in conflict management in healthcare is highlighted in studies from both the emergency department and operating rooms, where continued negative conflicts can lead to high staff turnover, and thus, loss of experiential knowledge that could affect patient outcomes [15, 16]. While many health professions’ curricula focus on interprofessional communication, there are few that address conflict resolution specifically [17–20].

Complicating matters further, there are various conflict resolution styles, such as competing, collaborating, compromising, accommodating, and avoiding [21, 22]. These styles tend to vary by personality [23] and, perhaps, even by profession [24, 25] or discipline [26]. Various styles may be preferable to certain circumstances or environments [22–24]. To our knowledge, there is no data on conflict resolution styles of medical students or on conflict resolution styles across different specialties in the United States.

Conflict resolution simulation could help graduating medical students become versatile in dealing with real world conflict in healthcare facilities. By understanding the different types of conflict resolution styles that exist in students and their skill with conflict resolution, we can better tailor training to improve student’s skills with resolving conflict prior to residency.

We created a conflict resolution simulation for graduating medical students as part of their transition to residency course [21]. The objectives of this study were to (1) evaluate conflict resolution styles amongst graduating medical students, (2) assess if conflict resolution skills as judged by trained faculty coaches were impacted by students knowing their conflict resolution style prior to the session or by other personal characteristics (e.g. intended specialty, gender, and/or under-represented minority (URM) status) and (3) assess the value to students of practicing conflict resolution skills while considering one’s predominant conflict resolution style.

## Methods

### Research study design

This study was a prospective, group randomized, single-blind quasi experimental trial involving 108 students enrolled in a single transition to residency course and their coaches at Duke University from January- April of 2017. This method was chosen to allow both groups of students the same educational content, taken in a different sequence. Group randomization occurred for purposes of scheduling in the simulation center. Only faculty evaluators were blinded to sequencing of the educational content.

### Conflict resolution curriculum in capstone

Duke University School of Medicine has a mandatory, longitudinal transition to residency course for fourth-year medical students. Individual students are assigned faculty coaches (1 faculty: 8 students) at the start of the course. Coaches provide individual feedback to students across several exercises, such as a discharge summary, informed consent, managing difficult conversations, handoffs, simulated paging, triage, and high-fidelity simulation. In 2017, conflict resolution was added to this curriculum. A full description of the conflict resolution curriculum, including students pre-work, description of the simulation and training of actors, post-work and assessment rubric has been previously published [27]. In brief, the conflict resolution session is mandatory for all students and consists of three parts: pre-work, a standardized patient (SP) simulation encounter, and post-work. During prework, students review videos of conflict resolution, reflect on their own conflict resolution styles and complete a simulated conflict with another healthcare provider. In this conflict, an intern and nurse must negotiate how to obtain additional intravenous access after patient loses access. The intern is told in advance to try not to place a central line in order to preserve opportunity for hemodialysis access in the future, while the nurse (an actor) wishes for central access to minimize phlebotomy (“sticks”) for the patient. Simulation is video recorded.

After simulation, the student and the student’s coach review the student’s video and assess the student’s conflict resolution skills using a previously described rubric [27]. This rubric included skills with negotiation and generalizable communication skills and a summative evaluation of entrustability.

The negotiation score was developed by finding overlap from conflict resolution skills in the list of entrustable behaviors for teamwork published by the American Association of Medical Colleges [28] with conflict resolution skills published in the business literature. Overlap occurred in four areas: identifying the problem, breaking the problem into pieces, agreeing on a common goal, and assigning responsibility. The negotiation score was the sum of scores from four 10-point assessments on the student’s ability to: (1) identify/acknowledge there was a problem, (2) break the problem into smaller pieces, (3) acknowledge a shared goal, and (4) summarize a plan that worked for both parties. Each ranking had a descriptor for “0” and for “10”. The maximum amount of points for this section was 40.

Generalizable communication skills were defined in concert with clinical skills director and included skills students had been assessed on in prior standardized patient encounters including: (1) ability to listen, (2) ability to acknowledge concerns, (3) ability to ask questions effectively, (4) appropriate body language, (5) emotional intelligence, and (6) patient-centered conversation. The maximum amount of points for this section, called the emotional quotient score, was the sum of scores from six 100-point assessments for a total of 600.

Entrustability was determined based on the Association of American Medical Colleges (AAMC)’s Core Entrustable Professional Activities (EPA) for Entering Residency #9: Collaborate as a member of an interprofessional team [28]. To develop agreement on entrustment, faculty reviewed pilot videos of student performance with two different standardized patients and agreed on behaviors that would result in pre-entrustment. For example, never soliciting the opinion of the bedside nurse, not asking them their understanding of the problem, or never taking time to listen to the nurse. Inter-rater reliability was not determined prior to the exercise. Instead, post-hoc analysis was completed to determine if any faculty rater was an outlier in this, or in any of the other transition to residency simulations.

**Fig. 1:**
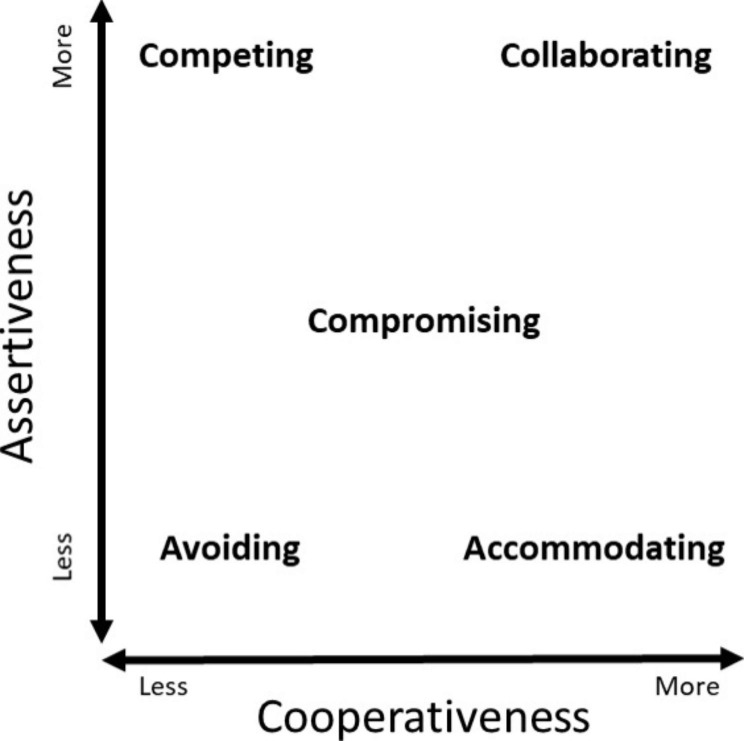
The five-conflict resolution styles as described by the TKI on scales of assertiveness and cooperativeness. Figure adapted from TKI [29]

To evaluate if awareness of conflict resolution style effected conflict resolution performance, we randomized the groups of students to taking the Thomas-Kilmann Instrument (TKI) before the simulation or after the simulation; these will henceforth be referred to as the “before” and “after” groups, respectively. The TKI is a validated and widely used tool to describe and study behavior in conflict scenarios [21, 22, 29]. Based on a series of questions, the instrument determines the respondent’s primary conflict style(s) [29]. The five described styles are accommodating, avoiding, collaborating, competing, and compromising. All differ on the scales of assertiveness and cooperativeness as shown in Fig. 1 [29].

**Fig. 2:**
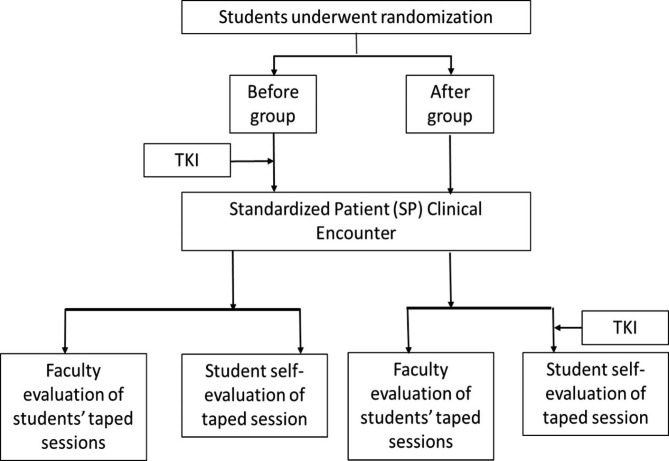
Students were randomized into before and after groups. In the before group, the TKI was taken prior to the SP encounter. In the after group, the TKI was taken after the SP encounter, but prior to the self-evaluation

Student groups were randomized by flip of coin to the “before” or “after” group. Randomization occurred in groups to facilitate timing of simulation with standardized patients. Groups randomized to the completing the TKI after their session needed longer time in the simulation space, thus impacting scheduling of students in that space. While the clinical skills lab director was aware of randomization for the purpose of scheduling students, this randomization did not affect the duration of time for the SP encounter. SP and faculty coaches were blinded to the students’ groups. See Fig. 2 for the study procedure.

Students rated the value of the exercise in their postwork using 5-point Likert-scale (from not useful = 0 to extremely useful = 5) Students were asked specifically on the usefulness of the exercise in regards to (1) having information from the TKI prior to the SP encounter, (2) learning the background to conflict resolution, (3) reviewing the pre-work videos, and (4) self-assessing their own performance in conflict resolution by viewing a video of their encounter.

The potential impact potential impact of gender, under-represented minority status, and intended specialty on faculty determinations of entrustability were assessed after course completion and after final grades for the pass/fail course were issued.

### Data collection

All prework, postwork, and faculty assessment were completed in Qualtrics, an online survey tool. Data was downloaded in aggregate form to a spreadsheet by the course director after course completion. Each student was issued a unique survey for the Thomas-Kilman Instrument (TKI), which was administered by Kilmann Diagnostics. Data was provided to the course director after course completion by Kilmann Diagnostics. Gender, race, and ethnicity were reported by students in their admissions materials, and shared from the School of Medicine to the biostatisticians of this study. Students intended field of practice was reported by students in the graduation bulletin. This data was extracted by the course director onto a spreadsheet. Specialties were identified as diagnosis-based (medicine, internal medicine, psychiatry, pediatric, neurology, community and family medicine), procedure-based (surgery, anesthesiology, emergency medicine, dermatology, and obstetrics and gynecology), or other (pathology, radiology, radiation oncology, physical medicine and rehabilitation, and consulting). Data from these sources were electronically aggregated/matched for each student by the biostatitiscians onto one spreadsheet. All materials for this study were stored in an online, secured file with access limited to the authors of this paper.

### Statistical analysis

Students were randomized into two groups: those who took the TKI before SP encounter and those who took TKI after the SP encounter. Conflict resolution skills were assessed by the negotiation score, emotional quotient score, and entrustability. Conflict resolutions styles were reported from the TKI. Other explanatory variables included gender, URM status, and intended field of practice.

Continuous variables were summarized with the median, and 25th and 75th percentiles, and categorical variables were summarized with frequencies and percentages. The outcomes were compared between groups using the Wilcoxon rank-sum test for scores and chi-square or Fisher’s exact test where appropriate for survey questions with categorical values. All tests were two-sided and the level of statistical significance was set to 0.05 without adjusting for multiple comparison. Statistical analyses were performed using SAS 9.4 (SAS Institute, Cary, NC).

#### Participants and ethical considerations

All students enrolled in the transition to residency course were required to complete the simulation exercise as part of the mandatory transition to residency course. Students received all components of the curriculum (just in a different order), and evaluations of student performance were *not* used to assign a grade to the student. Instead, students were offered the opportunity to repeat the exercise if they wished after watching their own videos and/or receiving feedback from coaches. This study was reviewed by the Duke University School of Medicine Institutional Review Board and assigned exempt from further review.

## Results

One-hundred and eight students participated in the conflict resolution course. There were 67 students randomized into the before group. There were 41 students randomized into the after group. The groups had similar distributions of age, gender, URM status, or specialty. Table 1 summarizes the students’ characteristics.

**Table 1:** Student Characteristics

Student Characteristics	TKI After (n = 41)	TKI Before (n = 67)	Total (n = 108)
**Age**			
Median (25th, 75th percentiles)	27 (26, 30)	27 (26, 28)	27 (26, 29)
Not reported	6	10	16
**Gender**			
Male	19 (46.3%)	31 (46.3%)	50 (46.3%)
Female	14 (34.1%)	27 (40.3%)	41 (38%)
Other or Unknown	8 (19.5%)	9 (13.4%)	17 (15.7%)
**Under-represented minority** **(African/Native American, Hispanic, Latino)**			
Yes	6 (14.6%)	9 (13.4%)	15 (13.9%)
No	24 (58.5%)	43 (64.1%)	67 (62.0%)
Other or prefer not to answer	11 (26.8%)	15 (22.4%)	26 (24.1%)
Specialty*			
Diagnosis-based specialty	15 (36.6%)	23 (34.3%)	38 (35.2%)
Procedure-based specialty	18 (43.9%)	34 (50.7%)	52 (48.1%)
Other or unknown	8 (19.5%)	10 (14.9%)	18 (16.7%)

* Diagnosis-based specialties included internal medicine, pediatrics, family medicine, psychiatry, and neurology. Procedure-based specialties included surgical specialties, anesthesia, emergency medicine, dermatology, and obstetrics and gynecology. Other included pathology, radiation oncology, radiology, physical medicine and rehabilitation, and consulting

### Conflict resolution styles

Table 2 summaries the primary conflict resolution styles found amongst the students as reported from the TKI instrument itself. Students could have more than one primary style.

The most common styles were accommodating followed by compromising. As part of the curriculum, students also self-determined their primary conflict style prior to taking the TKI. Only 56.0% (n = 56/100) of students had accurate self-awareness of their predominant conflict resolution style as that determined by the TKI.

**Table 2:** Conflict Resolution Style Assessed by TKI

Conflict Resolution Modes	Total (N = 108)
Accommodating	37 (34.3%)
Compromising	20 (18.5%)
Avoiding	15 (13.9%)
Collaborating	15 (13.9%)
Competing	7 (6.5%)
Accommodating/Collaborating	2 (1.9%)
Collaborating/Avoiding	2 (1.9%)
Accommodating/Compromising	1 (0.9%)
Collaborating/Compromising	1 (0.9%)
* Eight patients did not report conflict resolution styles (7.4%)	

Note: A student could have more than one primary style

### Student characteristics and impact on conflict resolution skills

Table 3 summarizes differences between gender, URM status, and intended specialty in conflict resolution skills as evaluated by faculty at the time of reviewing each students’ videotaped encounter. Faculty gave higher emotional quotient scores to females (*p* = 0.02). Students pursuing diagnosis-based specialties had higher negotiation (*p* = 0.04) and higher emotional quotient (*p* = 0.006) scores.

**Table 3:** Conflict Resolution Skills in Relation to Gender, Intended Specialty, and URM Status

	Gender			Specialty				URM-status		
	Female	Male	*p*-value	Diagnosis-based	Procedure-based	Other	*p*-value	Yes	No	*p*-value
**Negotiation Score** Median (25th, 75th percentiles)	33.1 (28.1, 36.1)	30.2 (24.2, 33.7)	*0.14*	33.3 (28.2, 36.5)	29.6 (24.2, 33.1)	31.2 (24.5, 33.5)	***0.04***	31.3 (23.0, 35.4)	31.6 (25.0, 35.4)	*0.61*
**Emotional Quotient** Median (25th, 75th percentiles)	533.0 (505.0, 553.5)	510.0 (458.0, 540.0)	***0.02***	540.0 (505.0, 555.0)	507.0 (480.0, 530.0)	525.0 (471.0, 540.0)	***0.006***	506.5 (449.0, 544.0)	529.0 (492.0, 545.0)	*0.3*

* P-values were calculated from Kruskal-Wallis tests

### Entrustability and conflict resolution

Table 4 summarizes conflict resolution skills as judged by faculty after review of each students videotaped encounter (n = 98). There was no difference in conflict resolution skills between groups. The majority of students, 92.8% (n = 91), were evaluated as entrustable, and 7.2% (n = 7) as pre-entrustable. Among students evaluated as pre-entrustable, a common theme in faculty comments was the need to be more assertive.

**Table 4:** Conflict Resolution Skills and Entrustability between After and Before Groups

	After TKI (n = 41)	Before TKI (n = 67)	*p-*value
**Entrustability**			
Entrustable	35 (97.2%)	56 (90.3%)	0.26^1^
Pre-entrustable	1 (2.8%)	6 (9.7%)	
Not evaluated	5	5	
**Total Negotiation Score** **(Max 40)**			
Median (25th, 75th percentiles)	31.7 (28.2, 36.5)	31.3 (24.5, 34.9)	0.29^2^
Range	(13.7–39.0)	(8.2–40.0)	
Missing	2	0	
**Total Emotional Quotient Score (Max 600)**			
Median (25th, 75th percentiles)	529.5 (501.0, 545.0)	514.5 (479.0, 546.0)	0.58^2^
Range	(395.0-580.0)	(301.0-600.0)	
Missing	3	1	

^1^Fisher’s exact test. ^2^Wilcoxon rank-sum test

There were no significant differences in assessments of entrustment between coaches in this simulation or across any of the other transition to residency simulations (conflict resolution, informed consent, shared decisionmaking, triage and paging).

### Value of the exercise

A majority of students thought the TKI promoted self-reflection (81/108, 75.0%). A higher proportion of students who took the TKI *before* their encounter with the SP (65/67, 97.0%) found reviewing the pre-work videos useful than the group who took the TKI after the encounter.

## Discussion

The most common conflict resolution style amongst fourth-year medical students was accommodating. The accommodating style focuses on satisfying the other person’s needs or concerns over one’s own. While there is no “correct” primary conflict resolution style, execution of patient care may require an intern to advocate for and/or compromise more rather than simply giving in to the needs of the other healthcare professionals [25, 26, 30]. The “student” mindset most likely contributes to conflict styles focused on appeasing the other party; medical students are often the junior members of a medical team. A course that focuses on communication skills that foster use of different conflict resolution styles and using the most effective style for a situation or scenario may be useful for graduating medical students. For example, a high stakes code situation with an experienced clinician, a competing or collaborating style may be preferred. For an inexperienced trainee working with the same staff in an outpatient clinic setting, a compromising style may be better. For a patient with a trivial or inconsequential complaint, an avoidant style might be best. We could develop all of these scenarios and allow students opportunity to adapt to those situations. Alternatively, a student with a known conflict resolution style of competing could be asked to complete a simulation requiring an avoidant or compromising style.

Diagnosis-based specialties and female students had significantly higher negotiation and emotional quotient scores than procedure-based specialties and male students, respectively. Others have also described differences in communication by specialty and gender that impacted patient outcomes [31–34]. Ambady et al. showed that surgeons’ tone of voice during routine visits were associated with malpractice claims [35]. Levinson et al. showed that surgeons infrequently expressed empathy towards patients and focused more on operational information compared to internists [32, 33]. Female physicians have been shown to be better communicators through active partnership behaviors [34]. These differences in specialty and gender indicate that different curricula may be needed to target specific skills for different student groups. Perhaps a cross discipline simulation (surgery talking to medicine or vice versa) would require students to adapt to more or less negotiation skill.

We were delighted to find no differences in the faculty evaluation of URM and non-URM students especially in light of known racial disparities in student performance evaluations, grades, and membership in the Alpha Omega Alpha Honor Society [36–41]. We believe faculty development sessions to develop the evaluation rubric and to practice the rubric contributed to the lack of racial disparities in evaluation by coaches. As the number of medical schools assigning students to coaches increases, we believe that schools should undertake similar evaluations to look for unconscious bias in coach evaluation and to take the time for faculty development to set consistent expectations and to practice group evaluation.

Self-awareness of one’s conflict resolution style(s), as determined by TKI, was not associated with better conflict resolution skills as judged by standardized faculty. Though students reportedly found the TKI useful for their own self-reflection, the lack of impact on skill for this single exercise makes it difficult to justify the cost of administering this instrument ($35/per student).

Faculty assigned high levels of entrustment to our students for collaborating in an interprofessional team. The high rates of entrustability despite lack of prior explicit training, specifically in conflict resolution, may be related to a leadership thread in our medical school that includes patient-centeredness and emotional intelligence. Although the number was small, the students who were pre-entrustable may require more sessions with an SP to practice their skills or more feedback to recognize their weaknesses, such as, focusing on respectful assertion.

Our study had several limitations. While our sample size was adequate to look at the primary question of entrustability and types of conflict resolution styles, it might not have been adequate to analyze specialty, gender, and URM differences. We did not evaluate whether gender or specialty-concordance between student, SP, and faculty coach played any role in scoring. We had several female SPs for this study; this could have impacted the session and subsequent evaluation of the interaction, and in the future, we plan to include a male SP as well. Further, our faculty coaches are largely physicians (though we have one physical therapist and one nurse practitioner); we could have different members of the healthcare team assessing entrustability.

## Conclusions

The most prominent conflict resolution style among graduating medical students is accommodating. While knowing conflict resolution style in advance of a simulated conflict was of value to students, it did not influence their skill as judged by faculty coaches. Student gender and intended field of practice did effect conflict resolution skills. This may allow for future targeted curricula in conflict resolution.

## Electronic supplementary material

Below is the link to the electronic supplementary material.

Supplementary Material 1

## Data Availability

The datasets used and/or analysed during the current study are available from the corresponding author on reasonable request.

## Abbreviations

TKI: Thomas-Kilman Instrument
SP: Simulated Patient
NICU: Neonatual Intensice Care Unit
URM: Under-represented Minority
AAMC: Association of American Medical Colleges
EPA: Entrustable Professional Activities
