# Supplementary material for: Conflict resolution styles and skills and variation among medical students
Source: BMC Med Educ. 2023 Apr 14;23:246. doi: 10.1186/s12909-023-04228-x (PMC10103535; doi:10.1186/s12909-023-04228-x)
Supplement: Supplementary file 1 — Supplementary Material 1 [file 12909_2023_4228_MOESM1_ESM.docx]

**Capstone 2017 Conflict Resolution Student Evaluation of Thomas-Kilmann Instrument (TKI)**

1. Did you complete the TKI during the pre-work?
   1. Yes
   2. No

If yes to #1, the following questions will follow:

1. How would you rate the utility of having this information prior to the encounter with the nurse?
   1. Not useful
   2. A little useful
   3. Moderately useful
   4. Very useful
   5. Extremely useful
2. If c-e chosen for #2: Why was it useful?
3. If a or b chosen for #2: Why do you think it was not that useful?

If no to #1, the following questions will follow:

1. Please take a moment to complete the TKI and then return to the survey.
2. What was your conflict resolution style?
3. Would knowing this style have helped you with the standardized patient case? Why or why not?

For all:

1. What best described your self-reflection on conflict negotiation style/mode compared to the data that the TKI provided?
   1. My prediction of my conflict negotiation/resolution style from the pre-work was the same as the TKI.
   2. My prediction of my conflict negotiation/resolution style from the pre-work differed from the TKI.
   3. I can’t remember.
2. With regard to the rest of the pre-work, please rate how useful the following were to you:
   1. Learning the background to conflict resolution
      1. Not useful
      2. A little useful
      3. Moderately useful
      4. Very useful
      5. Extremely useful
   2. Reviewing the videos
      1. Not useful
      2. A little useful
      3. Moderately useful
      4. Very useful
      5. Extremely useful
   3. Specifically looking for behaviors within the videos
      1. Not useful
      2. A little useful
      3. Moderately useful
      4. Very useful
      5. Extremely useful
